# New insights into the cardiorespiratory physiology of weaned southern elephant seals (*Mirounga leonina*)

**DOI:** 10.1093/conphys/cov049

**Published:** 2015-12-08

**Authors:** Cloe R Cummings, Mary-Anne Lea, Margaret G Morrice, Simon Wotherspoon, Mark A Hindell

**Affiliations:** af1 Institute for Marine and Antarctic Studies, University of Tasmania, Hobart, TAS 7004, Australia; af2 School of Life and Environmental Sciences, Deakin University, Warrnambool, VIC 3280, Australia

**Keywords:** Chronobiology, circadian rhythms, generalized additive models, threshold physiological response

## Abstract

Southern elephant seal (*Mirounga leonina*) pups must strike a balance between conserving energy during their post-weaning fast and simultaneously developing diving abilities to attain nutritional independence. Little is known about environmental influences on cardiorespiratory patterns, hence energy use, throughout the 6 week fast. Continuous heart rates were recorded for free-ranging, newly weaned southern elephant seals using heart rate time–depth recorders for 5–9 days at Sub-Antarctic Macquarie Island, during October 1994 (*n* = 1), 1995 (*n* = 4) and 1996 (*n* = 1). Daytime observations of respiration and behaviour were made throughout. We present the first instance of synchronous heart rate traces recorded simultaneously for individual weaners. Generalized additive models revealed that a sinusoidal pattern of diurnal heart rate elevation and nocturnal depression was evident in all seals and, on at least one occasion, a conspicuous break in this pattern coincided with an extreme cold weather event. Seals in this study were capable of considerable cardiorespiratory control and regularly demonstrated bradycardia during periods of resting apnoea. Apnoeic duration ranged from 33 to 291 s (mean 134 s). Apnoeic heart rates (mean 67 ± 15 beats min^−1^, range 40–114 beats min^−1^) were on average 19.7% lower than those exhibited during periods of eupnoea (mean 83 ± 15 beats min^−1^, range 44–124 beats min^−1^). The early development of the cardiorespiratory response is characterized by arrhythmic heart and respiration rates. The strong temporal patterns observed are being driven by the opposing requirements of maximizing time spent fasting in order to develop diving capabilities and of maximizing departure mass. This pilot study has highlighted a potentially large effect of ambient weather conditions on newly weaned southern elephant seal cardiorespiratory activity. Given the increasing westerlies and more erratic and increasing storminess associated with the Southern Annular Mode predicted in the Southern Ocean, the patterns observed here warrant further investigation.

## Teaser

We present the first continuous, multi-day heart rate recordings of newly weaned, wild southern elephant seals. The effects of circadian periodicity and weather conditions on heart rate are examined. Changing climate regimes may affect the energetic requirements experienced during, and thus the energetic trade-off that concludes, the post-weaning fast.

## Introduction

Diving mammals exhibit unique cardiorespiratory traits to cope with the extreme physiological demands of diving (Butler and Jones, 1997; Mottishaw *et al.*, 1999; Davis *et al.*, 2004). One such adaptation is arrhythmic breathing composed of alternating periods of breath holding (apnoea) with associated depression of heart rate (bradycardia) and regular breathing (eupnoea; Williams, 1999, Williams *et al.*, 1999). In pinnipeds, this characteristic cardiorespiratory pattern occurs not only at sea but also on land (Castellini, 1996; Andrews *et al.*, 1997; Falabella *et al.*, 1999). Analogous ontogenetic aspects of the development of terrestrial apnoea and diving capacity suggest that these traits share a common physiological foundation (Blackwell and Boeuf, 1993; Boily and Lavigne, 1996; Castellini, 1996; Andrews, *et al.*, 1997). They are thought to represent a dual adaptation for enabling protracted periods of submersion whilst foraging at sea, as well as for conserving water and energy during fasting life-history phases on land (Blackwell and Boeuf, 1993; Mottishaw, *et al.*, 1999; Lester and Costa, 2006).

Elephant seals (genus *Mirounga*) are archetypal arrhythmic breathers. Adult individuals are typically submerged for 80–95% of the 8–10 months they spend annually at sea and are therefore highly adapted for a life where overall time spent underwater is essentially comparable to (and throughout their lifetime often exceeds) time spent on land (McConnell *et al.*, 1992; Blackwell and Boeuf, 1993; Andrews *et al.*, 1997). They are exceptionally proficient divers and regularly exceed their theoretical aerobic dive limit with no evidence of anaerobic metabolism (Hindell *et al.*, 1992; McConnell *et al.*, 1992; Davis and Weihs, 2007). The genus also has the most profoundly arrhythmic terrestrial respiratory pattern of any seal, with apnoeic periods lasting up to several minutes (Blackwell and Boeuf, 1993; Andrews *et al.*, 1997; Falabella *et al.*, 1999).

Elephant seals undergo rapid physiological and behavioural developments between birth and their departure for the first foraging trips to sea. This phase is composed of a 3–4 week suckling period followed by a 5–8 week post-weaning fast (McMahon *et al.*, 2000; Tift *et al.*, 2013). During this time, the duration and frequency of apnoea increase as a function of age, as does the magnitude, regularity and temporal refinement of the bradycardic response (Blackwell and Boeuf, 1993; Falabella, *et al.*, 1999). Soon after the post-weaning fast, diving capabilities approach adult levels (Reiter *et al.*, 1978; Arnbom *et al.*, 1993; Butler, 1993; Boeuf *et al.*, 1996; Modig *et al.*, 1997). As heavier pups tend to fast for longer than lighter pups, the timing of weaner departure from the colony is believed to represent a trade-off between energy reserves that provide sustenance and thermoprotection for the coming months at sea and developmental state (Arnbom *et al.*, 1993; Noren, 2002). Juvenile mortality in the first year at sea can be very high in elephant seals [54–98% (Hindell, 1991); 35% (McMahon *et al.*, 1999); 50% (Reiter *et al.*, 1978)], and thus the nature of the trade-off at the end of the post-weaning fast may be a crucial factor in determining first-year survival.

The energetic requirements of fasting may not be static or predetermined over time. The relationship between heart rate and metabolism has been established for several pinnipeds and may be influenced by extrinsic weather conditions and circadian periodicity in environmental factors such as temperature and light (Butler, 1993; Boyd *et al.*, 1999; Butler *et al.*, 2004). Behavioural adjustments can also influence energetic requirements in a variety of mammalian species (MacArthur *et al.*, 1979; Arnold *et al.*, 2004; Ochoa-Acuna *et al.*, 2009). Psychological stress, for example from perturbing environmental conditions, has also been known to elicit increases in heart rate (MacArthur *et al.*, 1979; Weimerskirch *et al.*, 2002; Martineau, 2007). Noren (2002) showed that energetic costs vary for northern elephant seal weaners in different environmental conditions in the laboratory. As yet, few studies have attempted to record continuous heart rate in free-ranging elephant seals on land (Castellini *et al.*, 1994; Andrews *et al.*, 1997). Thus, it may be not only body condition at weaning but also the environmental conditions and energetic requirements experienced throughout the post-weaning fast that have a bearing on first-year survival in *M. leonina* (Falabella *et al.*, 1999; McMahon, *et al.*, 2000).

Southern elephant seals are a major consumer of Southern Ocean resources (Bailleul *et al.*, 2007; McIntyre *et al.*, 2011; Walters *et al.*, 2014), which have experienced a significant and prolonged population level decline since the 1950s (Hindell, 1991; McMahon *et al.*, 2005). The climate at Macquarie Island has undergone a clear shift since the 1970s, with increases in wind, precipitation and cyclonic events (Adams, 2009). If ambient weather conditions do influence the energetic requirements of animals throughout the post-weaning fast, changing weather norms may have follow-on consequences for survivorship during the first year at sea. Quantifying such ecophysiological relationships and incorporating them into ecological models will enable more refined predictions of how animals will respond to changing environmental conditions (Cooke *et al.*, 2013). Thus, elucidating the significance and mechanics of cardiorespiratory patterns during a crucial developmental period (the post-weaning fast) is an important component of fully understanding the biology of *M. leonina* with a view to managing this species appropriately and to predicting its responses to changing environmental conditions in the future (Falabella *et al.*, 1999).

The purpose of our study was to examine cardiorespiratory control in known-age, free-ranging, weaned southern elephant seals and the factors that might influence this pattern and to consider how this might contribute to possible energetic savings that could improve survivorship in the first year. Our specific aims were as follows: (i) to examine the relationships between heart rate and respiration (apnoeic and eupnoeic periods); (ii) to quantify temporal trends in heart rate; and (iii) to evaluate possible extrinsic influences on heart rate in weaned southern elephant seals.

## Materials and methods

### Study site

The study took place over 3 years on the (sheltered) east and (exposed) west coasts of the isthmus at Macquarie Island (54°35′S, 158°55′E). Weaned southern elephant seals of similar age (3–5 weeks) were randomly selected from a group of 400–800 tagged animals in each year (1994, *n* = 1; 1995, *n* = 4; 1996, *n* = 1; Table 1). The weight of none of the study animals fell below one standard deviation of the average weaning mass for their respective sexes for seals from Macquarie Island; it is therefore assumed that the study animals were healthy and fairly representative of the average population (Battaglia and Valencia, 1997).

**Table 1:** Summary of location, sex, birth date, age at weaning, mass at weaning, age at deployment, predicted mass at deployment, placement of heart rate electrodes and the deployment duration of the study animals

Seal	Site	Sex	Birth date	Age at weaning (days)	Wean mass (kg)	Age at deployment (days)	Deployment mass (kg)^a^	Electrode placement	Deployment duration (days)
94w2	W	F	18 Sept. 1994	24	124	30	118	DV	7.6
95160	W	F	17 Sept. 1995	24	98	25	97	DV	6.5
95161	E	M	22 Sept. 1995	21	134	26	128	D	8.7
95162	W	F	14 Sept. 1995	24	128	27	124	DV	5.4
95163	E	F	22 Sept. 1995	25	144	26	143	D	10.7
96161	E	F	20 Sept. 1996	27	127	32	121	DV	7.7

Abbreviations: D, dorsal; DV, dorsoventral; E, east coast; F, female; M, male; and W, west coast. ^a^Deployment mass was calculated with mass-specific mass-loss equations post-weaning, as follows: males 9.30 ± 0.7 g kg^−1^ day^−1^ and females 10.2 ± 0.68 g kg^−1^ day^−1^ (Arnbom *et al.*, 1993).

### Device deployment

All seals were captured manually and anaesthetized with Zoletil 100 (as per Hindell and Lea, 1998). Mk 3e heart rate time–depth recorder (HR-TDR) and heart rate processor/transmitter (H2) units with external electrodes (Wildlife Computers; Webb *et al.*, 1998) were attached to the apical region of the dorsal midline of each seal using Cieba Geigy K268 epoxy resin (as per Hindell and Lea, 1998). Two placement methods were used for the external electrodes: (i) both electrodes situated on the dorsal (D) midline (10 cm anterior to the heart rate unit and 20 cm posterior); and (ii) dorsoventral (DV) placement (i.e. one electrode placed 10 cm anterior of the heart rate unit and the second on the ventral surface over the heart; Thompson and Fedak, 1993; Hindell and Lea, 1998).

A Polar^®^ heart rate watch transceiver was held 20 cm from the HR-TDR to confirm that units were transmitting properly. Heart rate values registered by the Polar^®^ heart rate watch were compared with the heart rate as measured by stethoscope to test the accuracy of the units prior to deployment. No discrepancies were found between the two methods. It is assumed that the heart rate units did not significantly alter the normal activity of the seals. A VHF radio transmitter (Sirtrack^®^) was also fitted to the dorsal surface of each animal to facilitate re-location of seals after deployment.

The HR-TDRs were programmed to record the number of heart beats during each 30 s sampling period (resolution, ±2 beats min^−1^) in wet and dry conditions (as per Hindell and Lea, 1998). This sampling interval was required to test the HR-TDRs prior to deployment on free-ranging adult female southern elephant seals during their post-breeding foraging trip (see Hindell and Lea, 1998), which recorded at sea the heart rate over 50 days. The sampling interval therefore had limitations for this shorter-term land-based study in accurately determining mean heart rate. Hindell and Lea (1998) observed in an adult female elephant seal that during short surface intervals the heart rate was underestimated by 10–15%, whereas the heart rate during dives of 10 min duration (or 20 sampling intervals) was not influenced by errors associated with transition periods. Therefore, in the present study the heart rate may be overestimated during short apnoeic events and underestimated during short eupnoeic events. This would result in the difference in heart rate being minimized between events, and so gives greater emphasis to any statistically significant results.

### Behavioural observations

Daytime behavioural observations were recorded in conjunction with heart rate during several observation sessions throughout the study for all animals excluding 96161 (*n* = 5). Observations were done by a single trained observer out of view of the seal to prevent disturbance, but within sight of the seal's nostrils at all times to record changes in respiratory rates. An apnoea was defined as an expiratory pause of more than 30 s terminated by an inspiration (Falabella *et al.*, 1999). A eupnoea was defined as the respiratory period between successive apnoeas. Only recordings of animals that were undisturbed by humans were included in analyses. Swimming behaviours were not evident in these seals because of their young age and early developmental stage.

### Weather

Data for meteorological conditions [precipitation (in millimetres), dry-bulb temperature (in degrees Celsius), dewpoint temperature (in degrees Celsius), relative humidity (as a percentage) and wind speed (in kilometres per hour)] for the study period were obtained from the Australian Bureau of Meteorology (BOM) archives at the finest available resolution, namely 3 h sampling intervals. The wind chill factor (apparent temperature) was derived using the following equation (Steadman, 1994):

$$\mathrm{AT}=T_{a}+0.33e-0.70ws-4.00,$$

where *T_a_* is the dry bulb temperature (in degrees Celsius), *e* the water vapour pressure (in hectopascals) and *ws* the wind speed (in metres per second) at an elevation of 10 m above sea level.

The water vapour pressure is calculated as follows:

$$e=\left(\frac{rh}{100}\right)\;6.105\text{ exp}\left(\frac{17.27\cdot T_{a}}{237.7+T_{a}}\right),$$

where *T_a_* is the dry bulb temperature (in degrees Celsius), *rh* the relative humidity (as a percentage) and exp is the exponential function.

The west coast of the isthmus at Macquarie Island is more exposed to passing weather systems than the east coast. There is only one meteorological station on the island and so the data cannot differentiate the slight differences in severity in weather experienced between the coasts that would have occurred ‘on the ground’.

### Data analysis

The HR-TDR data were downloaded to a computer and transformed and analysed using custom-designed software after the recovery of each unit (Wildlife Computers Software). The first 6 h of data were removed to ensure that any effects of the anaesthetic or from disturbance due to handling were excluded from analyses. Daily solar elevation values were calculated for Macquarie Island using the elevation function in the tripEstimation package (Sumner and Wotherspoon, 2008) in R statistical software as per Lea *et al.* (2010). Positive elevation values were designated as ‘day’, values between 0 and −18° as ‘twilight’, and values lower than −18° as ‘night’ (Geoscience Australia, 2013).

To separate inter-individual from inter-annual effects, comparative heart rate analysis was done only for seals deployed in 1995 (*n* = 4). The difference between apnoeic and eupnoeic heart rates was assessed with Student's paired *t*-tests of heart rates from successive whole-apnoea and whole-eupnoea respiratory state pairs for each individual. Linear regression was used to test for relationships between apnoeic heart rate and duration of apnoea for individual seals.

Temporal patterns in heart rate across sites and individuals were compared by fitting generalized additive models (Wood, 2006) to the time series for the four individuals of similar age collected in October 1995, aggregated to 5 min intervals. Recurrent diurnal patterns were separated from longer-term trends by fitting both a smoothing spline in Julian day and a periodic spline in hour of day. Models that allowed these trends to vary by site and individual were fitted and compared by Akaike information criterion. To allow for serial correlation, model errors were assumed to have a first order auto-regressive (AR(1)) structure (Wood, 2006) and models were fitted by penalized quasi-likelihood with the mgcv package (Wood, 2011).

Statistical tests were run using R statistical software. All means are expressed ±SD and *P*-values as <0.05.

## Results

### Temporal trends in heart rate

**Figure 1:**
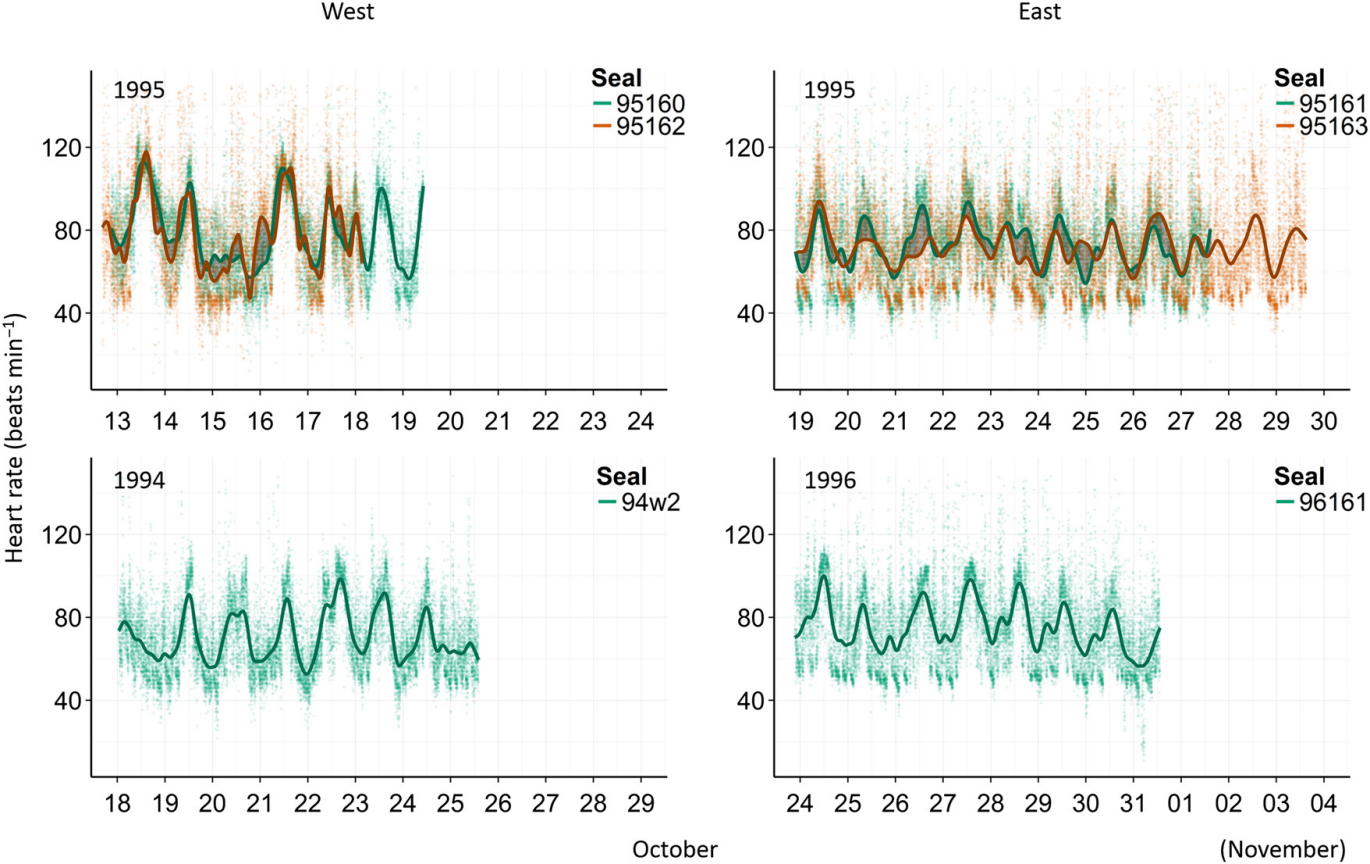
Raw data (within a limited heart rate range of 0–140 beats min^−1^) and smoothing splines with 45 degrees of freedom of multiday heart rate traces for all seals. Pairs from 1995 are displayed together in the top two panels, with differences in heart rate splines accentuated by grey shading (95160 and 95162, left; and 95161 and 95163, right). Year classes when only one individual was deployed are displayed in the bottom two panels (94w2 from 1994, left; and 96161 from 1996, right).

Continuous heart rates were recorded for six seals for periods ranging between 6.5 and 10.7 days (Table 1). Seals ranged in age from 25 to 32 days at deployment. All seals had the highest heart rate (and greatest variance) during the day, which decreased to a twilight or nocturnal minimum (Table 2 and Fig. 1). Over the entirety of all deployment periods in 1995, the mean heart rate amplitude was significantly higher on the west coast compared with the east (one-way ANOVA, *F*_1,90443_ = 1632, *P* < 0.05).

**Table 2:** Heart rate (in beats per minute) mean (±SD) and range for each seal, for each time-of-day bracket

	Day			Twilight			Night		
Seal	(mean ± SD)	Range	*n*	(mean ± SD)	Range	*n*	(mean ± SD)	Range	*n*
94w2	76 ± 17	28–148	8	65 ± 15	22–162	15	60 ± 14	26–148	8
95160	85 ± 21	22–206	7	70 ± 17	20–174	14	71 ± 17	20–170	7
95161	78 ± 19	2–226	9	67 ± 18	26–182	17	63 ± 17	18–160	9
95162	86 ± 31	0–252	5	67 ± 26	0–220	11	73 ± 24	0–210	6
95163	77 ± 21	0–252	11	68 ± 21	0–212	21	62 ± 19	0–220	11
96161	80 ± 19	0–206	8	71 ± 18	26–166	15	66 ± 16	32–156	8
Pooled	79 ± 21	0–252	8	68 ± 19	0–220	15	66 ± 19	0–220	8

*n* is the number of complete time-of-day brackets (full nights, days, or twilight periods) of data used to derive the mean and range of heart rates for that period, for that seal.

**Figure 2:**
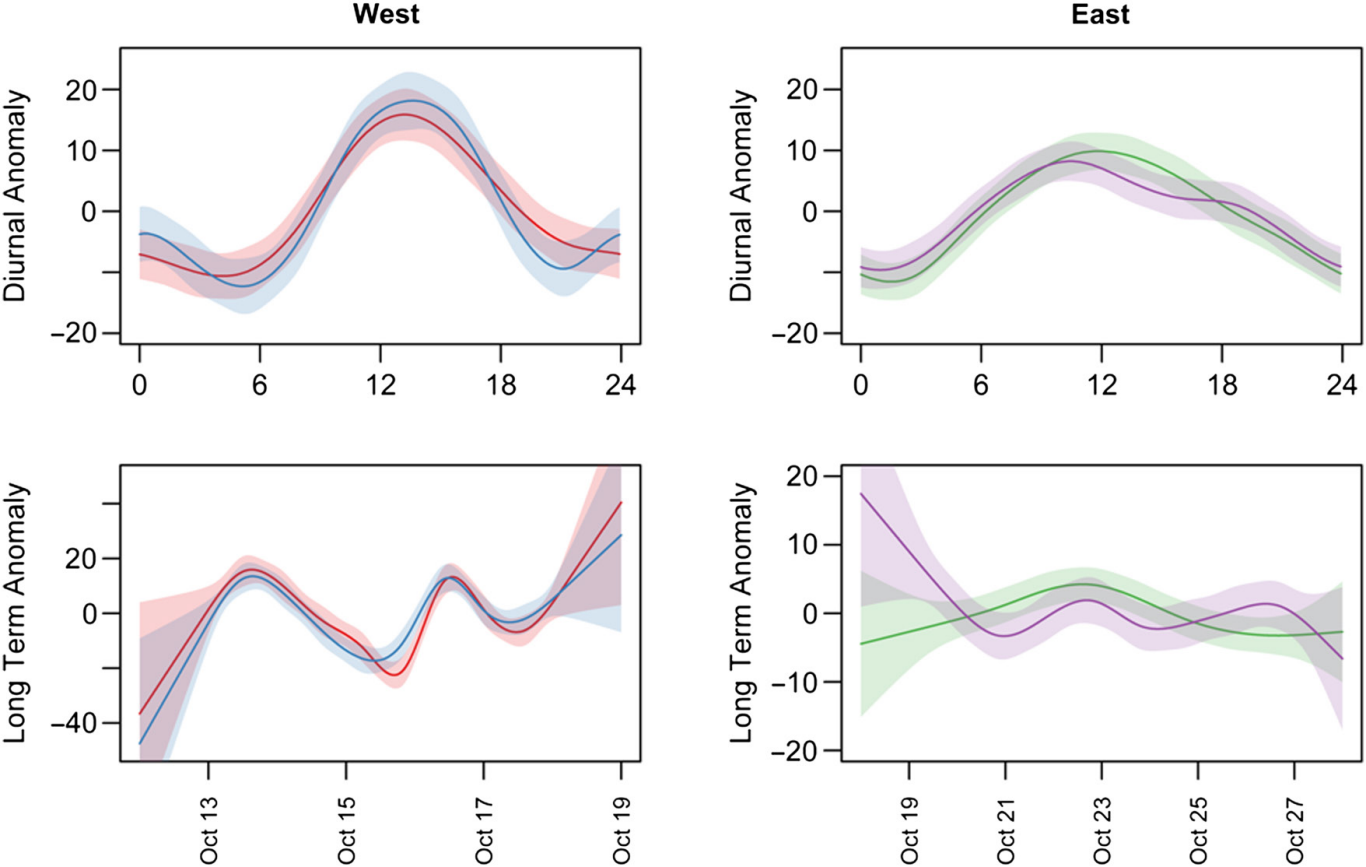
Smoothed temporal trends of individual weaners' heart rates with confidence intervals (diurnal in top panel and long term in bottom panels). Individuals are presented in site groups (west coast on the left; east coast on the right).

**Figure 3:**
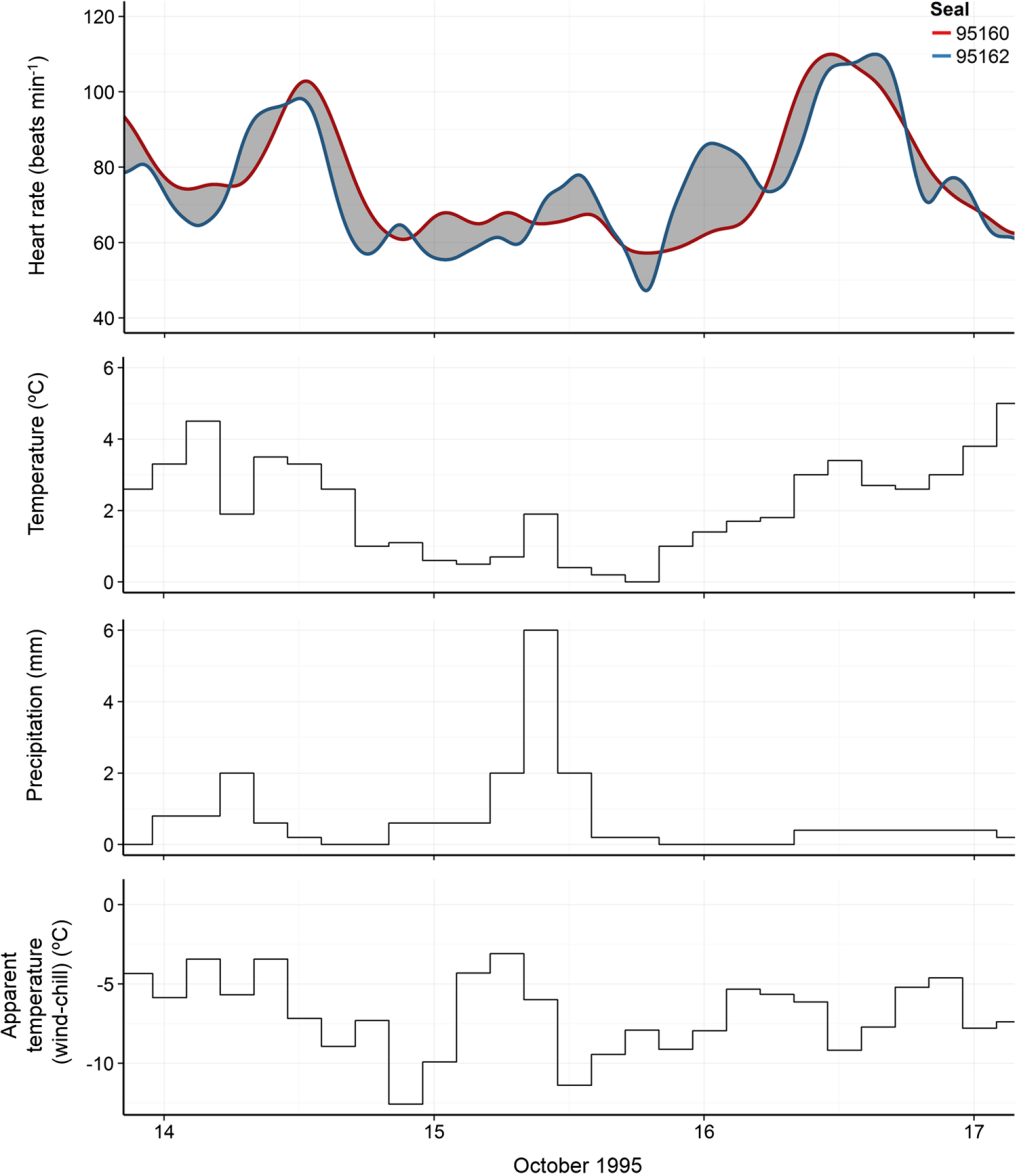
A closer inspection of the effect of an extreme cold weather event on 15 October 1995, on the heart rates of the two seals (95160 and 95162) deployed at the time. Raw heart rate data and smoothing splines with 45 degrees of freedom, displayed with corresponding ambient temperature, precipitation, and wind-chill conditions.

Generalized additive models of both east- and west-coast pairs of seals indicated a strong diurnal trend in heart rate patterns typified by higher levels during the day and lower rates at night (Fig. 2 and Tables 2 and 3). Over and above this diurnal trend, a long-term anomaly influence is evident in heartrates for the west-coast pair of seals. A negative anomaly on 15 October coincided with low temperatures, high wind chill and increased precipitation (Fig. 3).

**Table 3:** Akaike information criterion values of generalized additive models fitted to different permutations (pooled vs. individual) of diurnal and long-term temporal trends in heart rate data, fitted separately by site for the east- and west-coast weaners

Diurnal	Long term	West	East
Individual	Individual	23690.4	36811.0
Site	Individual	23669.0	36812.3
Individual	Site	23677.1	36802.9
Site	Site	23655.3	36805.1

### Weather and heart rate

The ‘wind-swept’ west coast of the isthmus at Macquarie Island is known to be generally more exposed to prevailing westerly weather systems than the relatively sheltered east coast (Selkirk *et al.*, 1990). Across the deployment periods in 1995, the average temperatures (west coast, 3.00°C; east coast, 2.81°C) and apparent temperatures (west coast, −6.03°C; east coast, −7.46°C) were comparable between sites. However, in addition to the probable difference in weather severity between coasts (masked by having only one weather station on the island), the average precipitation levels were considerably greater during the west-coast deployments relative to the east coast in 1995 [west coast, 0.87 ± 1.23 mm (3 h)^−1^ vs. east coast, 0.42 ± 0.56 mm (3 h)^−1^].

The pronounced depression of heart rates of the west-coast seal pair (95160 and 95162) on 15 October 1995 is of particular interest (Figs. 1 and 3). Unfortunately, no behavioural observations were made on this day; however, in several instances a mean drop in daily heart rate was associated with the co-occurrence of low apparent temperature (wind chill) and high precipitation.

### Cardiorespiratory control

Heart rates during apnoeic and eupnoeic events were significantly different for all four of the seals with enough observations to run the analysis (Table 4). The decrease (7.8–23.7%; Table 5) in heart rate from eupnoeic to apnoeic events ranged from 44 to 124 beats min^−1^ during eupnoeas (mean 83 ± 15 beats min^−1^) to from 40 to 114 beats min^−1^ (mean 67 ± 15 beats min^−1^) during apnoeas.

**Table 4:** Student's paired *t*-test results for difference between apnoeic and eupnoeic heart rates for four seals

Seal	*t*-stat	d.f.	*P*-value	Mean difference (beats min^−1^)
94w2	−9.7408	9	<0.05	17.86
95161	−7.99	27	<0.05	11.68
95162	−4.63	6	<0.05	14.06
95163	−8.80	13	<0.05	18.77

**Table 5:** Summary of means (±SD) and ranges for duration (in seconds) and heart rates (in beats per minute) for apnoeic and eupnoeic events for each seal, as well as percentage change (Δ) from average apnoeic to eupnoeic heart rates (final column)

			Respiratory parameters (s)				Heart rate (beats min^−1^)				
			AP		EU		AP		EU		
Seal	AP (*n*)	EU (*n*)	Δ (%)	Mean duration	Range	Mean duration	Range	Mean rate	Range	Mean rate	Range
94w2	11	10	175 ± 84	53–291	402 ± 663	89–2169	57 ± 10	42–96	75 ± 16	48–124	23.7
95160	2	1	97 ± 25	79–114	–	–	65 ± 9	56–82	71 ± 8	56–82	7.8
95161	33	29	126 ± 60	33–309	227 ± 202	76–826	63 ± 12	40–100	77 ± 9	44–102	17.3
95162	9	7	107 ± 65	54–245	173 ± 69	107–313	71 ± 14	46–100	84 ± 7	72–108	15.5
95163	18	15	139 ± 68	37–336	315 ± 479	60–1965	77 ± 14	50–114	98 ± 12	72–124	21.5
Pooled	73	62	134 ± 68	33–336	270 ± 377	60–2169	67 ± 15	40–114	83 ± 15	44–124	19.7

Abbreviations: AP, apnoea; and EU, eupnoea.

The apnoeas observed during the study (*n* = 73) ranged in duration from 33 to 336 s with a mean duration of 133.5 ± 67.8 s for five seals (Table 5). As a focus of the study was on capturing apnoeic heart rate, there may have been a bias towards capturing the shorter eupnoeas that occur between apnoeic events; however, full eupnoeas observed in this study (*n* = 62) ranged in duration from 60 to 2169 s with a mean duration of 270.3 ± 376.9 s for four seals. A weak inverse relationship between apnoeic heart rate and apnoeic duration was evident for one seal, 95161 (linear regression, *y* = −0.05*x* + 71.74, *F*_1,31_ = 8.003, multiple *r*^2^ = 0.21, *P* < 0.05). As this seal had double the number of successive respiratory state paired observations as the next highest individual, it is probable that small sample sizes substantially reduced the power to detect relationships in the remaining seals. Eupnoeic heart rate was also unrelated to the duration of eupnoea in all seals (*n* = 5).

**Figure 4:**
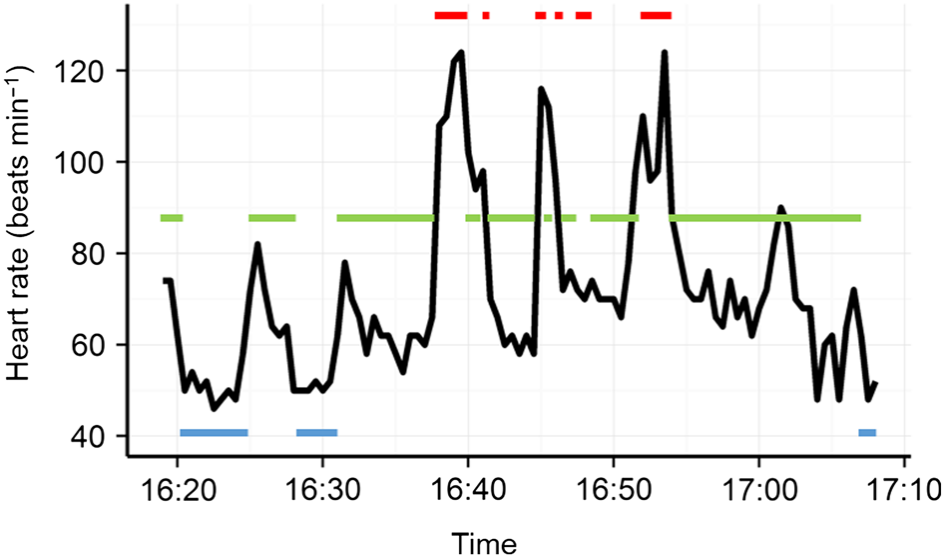
Sample activity level vs. heart rate for seal 94w2. Activity levels are indicated by horizontal coloured lines (active movement denoted by red; awake but sedentary by green; and sleep by blue).

Figure 4 illustrates the presence of bradycardia and simultaneous apnoeic events recorded by the HR-TDRs and simultaneous focal behavioural observations for one animal*.* Apnoeic bradycardia and inspirational tachycardia are both evident despite the limitations of the sampling intervals. The increase in heart rate during a short period of activity emphasizes the reduced heart rate recorded during apnoeic events. For each of the five animals for which behavioural observations were taken, mean heart rate increased as a function of increasing activity level (Table S1).

## Discussion

Highly variable heart rates are a fundamental physiological adaptation of southern elephant seals. Seen in this light, the synchronicity observed in this study between the heart rates of simultaneously deployed wild southern elephant seal weaners uncovers a new question that may prove central to understanding the energetics of the post-weaning fast. Given that individuals must have been performing different behaviours at least some of the time throughout the study, to what extent do environmental factors have a mediatory effect on the seals' heart rates?

### Temporal trends in heart rate

All weaners exhibited a daily pattern of higher diurnal heart rate and nocturnal decline (Fig. 2). Environmental variables that change with a 24 h periodicity, such as temperature and light, may directly influence an animal's chronobiological patterns in terms of metabolic activity or indirectly influence it by bringing about changes in activity. Although nocturnal behavioural observations were not done in this study, given the clear link between activity levels, respiratory state and heart rate seen in our animals, with (daytime) apnoeic events directly related to lower average heart rates (see Fig. 4), it is possible that there was an increase in the frequency of sleep-related apnoea during the night. Castellini *et al.* (1994) observed extreme bradycardia during slow-wave sleep in northern elephant seal pups. In both previous studies of southern elephant seal weaner behaviour, weaners were inactive for the vast majority (>90%) of time spent ashore (Modig *et al.*, 1997; Falabella and Campagna, 1999). Bartholomew (1954) observed that hauled-out northern elephant seals were predominately inactive, even more so during the night. In Patagonia, three weaners (aged >50 days) displayed relatively higher levels of nocturnal activity; however, in the same study the one animal of a comparable age (40 days old) to our weaners was more active during the day, consistent with the heart rate pattern we observed (Falabella and Campagna, 1999).

Decreased metabolism has been observed nocturnally and during sleep in mammals without habitual apnoea, and thus decreased nocturnal heart rates may also have occurred independently of apnoea frequency in our animals (Shapiro *et al.* 1984; Arnold et al., 2004). Indeed, nocturnal hypometabolism (with associated peripheral body shell cooling) has been posited as an adaptive energy-saving physiological process to explain the diurnal metabolic and heart rate patterns observed seasonally in red deer when exposed to severe weather conditions and limited food availability (Arnold *et al.*, 2004). It has been suggested that such a physiological response may be both well regulated and more common than previously described for endotherms living in extreme climates where energy conservation is paramount. Situational hypometabolism occurs in *M. leonina* during diving and apnoeic periods (Tift *et al.*, 2013), and the heart rate pattern observed in the present study suggests that it also occurs nightly during the post-weaning fast.

Numerous studies have demonstrated that, when properly calibrated, inter-individually pooled heart rate data are a good indicator of field metabolic rate in a variety of pinnipeds (Butler, 1993; Boyd *et al.*, 1999; Butler *et al.*, 2004) and other mammals (MacArthur *et al.*, 1979; Arnold *et al.*, 2004; Young *et al.*, 2011). Heart and metabolic rates have not been calibrated for *M. leonina* and thus the exact nature of their relationship is not known in this species. Further investigation into the cardiometabolic dynamic in species with extreme bradycardia, and especially *M. leonina*, is necessary to gain a full understanding of the implications of sustained elevated or depressed heart rate on the energetic requirement of this species. Nonetheless, whatever the mechanisms behind the regular nocturnal reduction in heart rate described in the present study, it serves several key functions of the post-weaning fast: reduced energy consumption; conservation of valuable metabolic water lost as vapour during respiration (Huntley *et al.*, 1984; Blackwell and Boeuf, 1993); and better net protein synthesis via concomitant reduction of body temperature (Shapiro *et al.*, 1984).

Seals equipped with HR-TDRs in pairs showed unexpectedly high synchronicity of heart rate trends over and above the general diurnal pattern, throughout their deployment periods (Figs. 1 and 3). Physiological and behavioural mechanisms operating on cardiac control can be complex and non-linear; numerous studies on heart rate patterns in a variety of species describe inherent levels of inter-individual variation among subjects (Boyd *et al.*, 1999; Arnold *et al.*, 2004; Butler *et al.*, 2004). The similarity of the 1995 pairs is therefore strongly suggestive of a high level of influence by environmental factors on heart rate trends in *M. leonina* weaners. Owing to the difference in sampling intervals between heart rate (30 s^−1^) and weather variables (3 h^−1^) and the likelihood of weaners responding to weather cues on a scale of minutes as opposed to hours, we can currently present only a broad interpretation of the relationship between weaners' heart rates and their ambient environment.

The depression of both 1995 west-coast weaners' heart rates on a single day only is of particular interest and might reflect a response to challenging weather conditions. This day coincided with high rainfall and low ambient and apparent temperatures. Reiter *et al.* (1978) observed that inter-individual space between weaned northern elephant seals within groups varied with ambient temperature; in warm weather, animals were spaced further apart, whereas on cold days animals huddled together. Unfortunately, no behavioural observations were made on this particular day for our weaners, but throughout the study the weaned seals were observed huddling on several occasions, with as many as six or seven seals synchronizing their breathing rates. Bouts of simultaneous apnoea would be halted as the seals commenced respiration one by one, after which time the respiration rates slowed and apnoeas recommenced. We hypothesise that there is some energy-saving advantage for newly weaned elephant seals, not yet proficient divers, to huddle for thermoregulatory reasons in response to both lower ambient temperatures at night and particularly severe weather systems during the day. If long periods of apnoea are regularly associated with huddling behaviour, lower overall heart and metabolic rates would be expected.

The degree of synchrony between heart rate traces is visibly higher in the west-coast pair compared with the east (Figs 1 and 2). The difference in weather between the east- and west-coast microclimates suggests that increasing severity of weather conditions triggers an increasingly uniform cardiorespiratory response among individuals. This probably occurs both where the weather directly affects the physiology of the animals and indirectly, where heart rate is mediated by behaviour. Weimerskirch *et al.* (2002) described a tipping point for an inverse relationship between wind chill and heart rate in wandering albatross when temperatures dropped below 0°C. Not only were heart rate traces more consistently synchronous on the west coast, but mean heart rate amplitude and variance were also higher than that of the east-coast pair. Stress is known to increase heart rate in multiple species (MacArthur *et al.*, 1979; Weimerskirch *et al.*, 2002). If the weather was consistently more extreme on the west coast, this may have induced a constant stress response in the animals. However, inter-individual and temporal differences in free-ranging heart rate patterns cannot be explained by any one variable.

### Heart rate and respiration

The findings of the present study, i.e. the first continuous multiday heart rate recordings of wild, free-ranging southern elephant seal weaners, contextualize existing descriptions of a number of physiological capabilities of newly weaned southern elephant seals. The breath-holding ability of newly weaned pups that we observed was similar to that found in other studies; the duration of apnoea (0.6–5.6 min, mean 2.2 ± 1.1 min) was slightly shorter than that reported for weaned southern elephant seals of a similar age in Patagonia (3.06 min; Falabella *et al.*, 1999) and for 25- to 60-day-old northern elephant seals (mean 4.0 ± 1.3 min; Blackwell and Boeuf, 1993). It was slightly less than half that of weaned southern elephant seal pups departing on their first trip to sea (mean dive duration 5.4 min; Hindell *et al.*, 1999) or for post-weaning northern elephant seals (5.9 min; Thorson and Le Boeuf, 1994). This provides further evidence that breath-holding ability increases as a function of both age and development (Castellini *et al.*, 1994; Andrews *et al.*, 1997).

Eupnoeic heart rate (mean 83 ± 15 beats min^−1^) dropped by an average 19.7% during apnoeic periods (mean 67 ± 15 beats min^−1^), comparable to that recorded in elephant seals of a similar age by Castellini *et al*. (1994). Falabella *et al.* (1999) reported a 28% decrease in eupnoeic to apnoeic heart rate in weaners of a similar age (24–40 days old); however, this finding came from a smaller sample of apnoeic and eupnoeic cycles and a colony at Península Valdés, where the ambient weather conditions were probably less challenging than at Macquarie Island. Andrews *et al.* (1997) observed a 31% decrease in apnoeic heart rate for juvenile elephant seals on land, and Jones *et al.* (1973) reported diving heart rates 31% lower than eupnoeic heart rates for juvenile harbour seals. The smaller decrease recorded in the present study may be representative of reduced cardiac control attributable to the young age of the newly weaned seals and/or a result of the lower resolution of heart rate in relationship to the number of sampling intervals per event as detailed in the Materials and methods.

The negative relationship between apnoea duration and heart rate for one seal in this study also suggests that cardiorespiratory control is relatively advanced in such young animals. The same relationship has been noted during diving in adult free-ranging grey seals (Thompson and Fedak, 1993; Hindell and Lea, 1998) and in juvenile northern elephant seals (Andrews *et al.*, 1997), although not on land. This difference may be explained by a progression in cardiac control with age. Hindell and Lea (1998) observed a threshold in diving duration of 13 min for an adult female beyond which heart rate declined linearly. A similar relationship is evident for juveniles at sea (Andrews *et al.*, 1997). Perhaps apnoea durations on land fall under this threshold for the juvenile seals.

### Conclusions

The physiological responses of animals to extrinsic factors are becoming increasingly relevant as climate regimes begin to shift. At Macquarie Island, the number of calm days per year has decreased markedly since the 1940s and annual precipitation has increased by 35%, trends which are consistent with an increase in the Southern Annular Mode index; a concomitant increase in the number of cyclonic events passing over the island since the early 1970s has also been observed (Adams, 2009). Studying the chronobiology of wild animals in the complexity and richness of their natural habitats is the next step in elucidating the adaptive significance of internal animal clocks. This is especially pertinent when predicting how environmental changes in the future will affect the phenology of important life-history events (Kronfeld-Schor *et al.*, 2013).

Southern elephant seals have undergone an extended population level decline at Macquarie Island, and it is likely that anthropogenic climate change will affect this species further. The onus on managers in the future will therefore be to mitigate against this by limiting the effects of other (more direct) stressors, such as potentially increasing interactions with expanding fisheries (Burton and van den Hoff, 2002). Altered energetic requirements experienced by animals during the post-weaning fast may compound the effects of other factors influencing first-year survival, such as maternal investment and prey availability. Thus the ecophysiological relationship describing the energetics of the post-weaning fast may become a useful indicator for informing conservation management plans for this species in the future.

There are very few studies of continuous heart rate in free-ranging pinnipeds during terrestrial life-history phases for any demographic group. Whether the patterns described in this study, especially regarding the susceptibility of heart rate to exogenous forces, will prove to have further implications for reproductive or moulting phases later in life is presently unknown. However, any sustained change in heart rate must necessarily equate to changes in energetic costs. This would thus impact upon the energetic trade-off that dictates the length, and therefore available period for development, of the post-weaning fast in *M. leonina* and consequent survivorship at sea. Clearly, more research into the behavioural and physiological mechanisms operating in young elephant seals is required to test many of the assumptions associated with the suggestions raised in this article. More extensive behavioural and respiratory observations, additional physiological variables, finer-scale meteorological observations, greater sample sizes, temporal crossover between deployments at different locations, the quantification of thermoneutral zone and relationship between heart and metabolic rates in this species will help in the future to illuminate these remaining questions. Nevertheless, it is evident that the cardiorespiratory patterns experienced during the post-weaning fast by southern elephant seals are not fixed but exhibit some control and may fluctuate in response to changing weather conditions.

## Supplementary material

Supplementary material is available at *Conservation Physiology* online.

## Funding

This work was supported by the Australian Research Council (grant no. A09332789) and the Antarctic Scientific Advisory Committee (grant no. 589) and was conducted under permits granted by Tasmanian National Parks and Wildlife Service and the Antarctic Animal Ethics and Ionising Radiation Committee.

## Supplementary Material

Supplementary Data
