# Supplementary material for: New insights into the cardiorespiratory physiology of weaned southern elephant seals (Mirounga leonina)
Source: Conserv Physiol. 2015 Dec 8;3(1):cov049. doi: 10.1093/conphys/cov049 (PMC4778465; doi:10.1093/conphys/cov049)
Supplement: Supplementary Data [file cov049supp.zip › cov049supp.docx]

Supplementary material

1. Mean heart rate ± SD (beats min^-1^) per behavioural state for each seal.

|  | **Behaviour** | | | |
| --- | --- | --- | --- | --- |
| **Seal** | *Apneic (n)* | *Resting (n)* | *Lying (n)* | *Moving (n)* |
| 94w2 | 57 ± 10 (11) | 71 ± 11 (10) | - | 103 ± 18 (6) |
| 95160 | 65 ± 9 (2) | - | 71 ± 8 (1) | - |
| 95161 | 64 ± 12 (33) | 77 ± 9 (29) | - | - |
| 95162 | 71 ± 14 (9) | 84 ± 7 (7) | - | - |
| 95163 | 77 ± 14 (18) | 97 ± 11 (15) | - | 115 ± 7 (2) |
